# Hospital Meetings, &C.

**Published:** 1898-04-16

**Authors:** 


### HOSPITAL MEETINGS, &C.

### MILLER HOSPITAL, GREENWICH.

The annual general meeting of governors, held on Wednesday, April 6th, in the board-room of the institution, was presided over by Mr. Edgar Sydney. The accounts show that the ordinary income for the year, including £385 14s. 9d. from the Greenwich Diamond Jubilee Fund, amounted to £3,517, and the ordinary expenditure to £3,505. Under the heading of extraordinary income appear legacies amounting to £190, the extraordinary expenditure consisting of investments amounting to £511 14s. 9d., £126 of which was derived from life subscriptions, and the balance received from the Greenwich Diamond Jubilee Fund towards the endowment of a "Victoria" bed. In referring to the finances, the Chairman observed that but for the fact that they had had to invest a certain sum, there would have been a balance on the right side. As it is a deficiency of £309 is shown. On this point Mr. Cox, who seconded the adoption of the report, said that the Jubilee year had been an exceptionally good one for the hospital; in fact, he never remembered its position at the end of the year being so satisfactory. During 1897 278 in-patients were treated, 6,429 accidents, 7,615 out-patients, 581 lying-in, 1,768 dental, and 777 home cases; and the report states that the year has been one of greater usefulness and benefit to the sick poor in the district of South-East London than any on record since the foundation of the institution. The question of extending the hospital is receiving the anxious consideration of the committee. The beds are, as a rule, fully occupied, and the Chairman stated that as a consequence serious accident cases have sometimes to be turned away with regret simply because there is no accommodation. An isolation ward is also badly needed. The difficulty in the way is the financial one, and strong appeals are to be made for the necessary funds to extend the hospital's usefulness. There were only seven governors present at the annual meeting, and the Chairman, remarking on this, said it was strange the attendance should be so small, seeing that 800 or 900 notices had been sent out. Under the circumstances they must take it that the governors generally were satisfied with the manner in which the charity is administered. Messrs. Haycraft and F. Miller were appointed trustees, and votes of thanks given to the staff of the hospital.

### CENTRAL LONDON THROAT AND EAR HOSPITAL.

A large number of governors and subscribers filled the board-room of the hospital on the occasion of the twenty-fifth annual meeting of the Central London Throat and Ear Hospital, which was held on Tuesday, the 5th inst., Captain Alfred Hutton (treasurer of the hospital) in the chair.

After a brief introductory speech by the Chairman, the Secretary read an abstract of the report, which, on this occasion, was for twenty months. It showed that 7,429 new out-patients had been treated in the year ended March 25th, 1897, involving 48,880 separate attendances. In the same period 299 patients had been admitted into the wards. The ordinary income of the hospital for the year 1896-7 amounted to £1,974, and the expenditure was £2,240. Legacies amounting to £190 were also received.

Sir Henry Burdett, K.C.B., in moving the adoption of the report, spoke at some length on the subject of special hospitals. He said that he had always been in principle opposed to special hospitals, as he had always felt that decentralisation in medical relief, so far as the unit hospital was concerned, must necessarily be expensive, and therefore, from his point of view, undesirable. He was, however, bound to say that in studying this question year by year, as he had had the privilege of doing now for more than thirty years, and in watching the development of hospitals both in London, in the provinces, and abroad, he had come to the conclusion that the true test of the work of an institution was the same as the true test of the work of an individual, and that both must be judged by results. He did not think that the public, nor even those who were most interested in general hospitals, quite appreciated the difference there was between special hospitals and special hospitals in London; for example, the great bulk of the special hospitals who were doing useful work in the metropolis had had an existence of at least twenty-five years, and he was of opinion that when an institution had existed for twenty-five years, and continued to prosper and to extend the area of its influence during all that time, there must bemer it in it, or it could never have continued to thrive. (Applause.) If they were there that day to argue whether or not it was desirable to set up special hospitals and general hospitals in a new city which had neither, he would be obliged to range himself in support of the proposal to establish by preference one large hospital with adequate and properly-administered special departments. As matters stood, however, knowing and realising the work which had been done by the older special hospitals, he was bound to say that they had justified their existence. They were, therefore, in the position to claim that they were entitled to public support, and, what was even more important still, to public confidence, and he had no doubt that, so far as the best special hospitals were concerned, they would tend to extend the area of their work. He was led to this conclusion by finding that during the last twenty-five years only twelve special hospitals had been established in London, of which seven were devoted to the relief of lying-in women and of children (several of which might be regarded as homes rather than hospitals), and that the remaining five, with one doubtful exception, were not wanted. That fact, however, had nothing to do with the position of a hospital like the Central London Throat and Ear Hospital, which had justified its existence, and was doing its work in a way which proved to anyone who would take the trouble to inquire, that it was necessary and good work. Work which was necessary, because it not only provided medical relief for the poor, but it afforded opportunities to members of the medical profession to make themselves familiar with the special classes of disease which it was established to treat. He referred in strong terms to a class of institutions mis-called hospitals, of which the Queen's Jubilee Hospital was a striking instance. Another matter which appealed to him very forcibly with regard to special hospitals was the fact that they seemed to have reached their maximum number of out-patients. This was not because the treatment given at special hospitals was worse, not because the means of relief were less than at general hospitals, nor yet because the popularity of special hospitals was less than it formerly was, because he found at that hospital there was increased efficiency, increased skill and care lavished upon the patients, and an improvement in the appliances which modern science had produced. The reason was that the inquiry system, which that hospital was one of the first to institute, had proved in practice by patient persistence an entire success. Speaking of the Central London Throat and Ear Hospital, he showed that so far as the patients were concerned, so far as London itself was concerned, having regard to the class of patients dealt with—chiefly the bread winners—and so far as the educational value to the medical profession of the work done in the hospital was concerned, there was in that hospital something of great practical utility and importance. It gave him real pleasure to testify to them, as a practical man who understood this subject, that in this institution they had a well organised, well conducted, thoroughly useful hospital, which was doing good work, and ought to have all the support it needed in order to secure that that work should be continued and maintained. (Applause.)

Mr. Lennox Browne seconded the adoption of the report, and, in doing so, said that an interesting fact was noticeable in carefully examining the figures relating to the patients who attended the hospital, and that was that affections of the throat seemed to affect men more than women. Whereas in general hospitals the proportion of men to women was 8 to 10, in the Central London Throat Hospital it was 10 to 9.

The report was adopted.

The Management Committee, the chairman, and the auditors were re-elected, and votes of thanks concluded the proceedings.

